# Global, regional, and national burden of Alzheimer’s disease and other dementias from 1990 to 2021: findings from the Global Burden of Disease Study 2021

**DOI:** 10.3389/fnagi.2025.1678212

**Published:** 2025-12-01

**Authors:** Yinglei Duan, Chongfang Han, Hua Zheng, Jing Yu, Min Luo

**Affiliations:** 1 Department of Anesthesiology, Shanxi Bethune Hospital, Shanxi Academy of Medical Sciences, Tongji Shanxi Hospital, Third Hospital of Shanxi Medical University, Taiyuan, Shanxi, China; 2 Department of Anesthesiology, Hubei Key Laboratory of Geriatric Anesthesia and Perioperative Brain Health, Wuhan Clinical Research Center for Geriatric Anesthesia, Tongji Hospital of Tongji Medical College, Huazhong University of Science and Technology, Wuhan, Hubei, China

**Keywords:** Alzheimer’s disease and other dementias, COVID-19, Global Burden of Disease Study, incidence, prevalence, disability-adjusted life years

## Abstract

**Background:**

As the global population continues to age, the growing number of elderly individuals has contributed to an increase in age-related conditions and illnesses, such as dementia, which places considerable financial and emotional pressure on both families and society. Alzheimer’s disease (AD), the most prevalent form of dementia among the elderly, significantly impacts their quality of life and ability to perform daily activities independently. Over the past three decades, the age-standardized mortality rate has declined and life expectancy has steadily increased worldwide. However, this long-term progress was disrupted by the COVID-19 pandemic, which reversed some of the gains made in life expectancy and contributed to a global decline. The pandemic has notably affected global health, exacerbating the prevalence of Alzheimer’s disease and other dementias (ADOD).

**Methods:**

In line with the analytical approach used in the Global Burden of Disease Study (GBD) 2021, we examined the counts and age-standardized rates (ASR) for the prevalence, incidence, mortality, and Disability-Adjusted Life Years (DALYs) associated with ADOD, categorized by region, country, sex, and age from 1990 to 2021. To assess temporal patterns over the study period, we calculated the estimated annual percentage changes (EAPCs).

**Results:**

From 1990 to 2021, both the number and age-standardized rates (ASR) of prevalence, incidence, mortality, and DALYs associated with ADOD showed a significant increase globally. Regionally, high-middle SDI regions had the highest ASPR at 766.2 per 100,000 (95% UI: 659.8, 879.6), with the most considerable rise, reflected in an EAPC of 0.21 (95% CI: 0.17, 0.25). In East Asia, the highest age-standardized incidence rate (ASIR) was reported, at 149.6 (95% UI: 129.6, 171.1) per 100,000 people, along with the greatest increase in ASIR, at 0.4 (95% UI: 0.33, 0.47) among the 21 GBD regions. On a national level, China experienced the greatest burden of ADOD, with the highest ASPR of 900.8 (95% UI: 770.9, 1043.2) and the highest ASIR of 151.5 (95% UI: 131.2, 173.3) per 100,000 people. The 80–84 age group exhibited the highest rates of prevalence, incidence, and DALYs, whereas the number of deaths in the 85–89 age group surpassed those in other age categories. Throughout all age groups, females experienced a higher burden of ADOD than males, regardless of the time or geographical location. The burden of ADOD reached its lowest point in 2019 but has increased steadily since then.

**Conclusion:**

In summary, this study highlights the global epidemiological trends of ADOD from 1990 to 2021, including the impact of the COVID-19 pandemic on it. As the population ages, ADOD has emerged as a significant public health concern worldwide. Although some regions have made progress in managing the burden of ADOD, most regions and countries still face a heavy disease burden. More effective prevention and treatment strategies are needed to alleviate the impact of ADOD. In particular, greater focus should be placed on women and the elderly.

## Introduction

Dementia is a syndrome predominantly affecting older individuals, encompassing various subtypes such as Alzheimer’s disease (AD), vascular dementia (VD), frontotemporal lobe degeneration (FTLD), dementia with Lewy bodies (DLB), Parkinson’s disease dementia (PDD), and mixed dementia (MD) (Livingston et al., 2020; Scheltens et al., 2021). Alzheimer’s disease and other dementias (ADOD) are a group of progressive neurodegenerative diseases that mainly affect memory, cognitive function, and behavior (Zhao et al., 2025). ADOD is mainly characterized by progressive cognitive impairment and behavioral damage, including memory disorders, cognitive disorders, executive dysfunction, behavioral changes, and is accompanied by mental disorder symptoms in most patients (Choi et al., 2020). Patients endure significant physical and psychological strain over extended periods, severely affecting their quality of life. This is accompanied by substantial economic burdens, as well as the strain on family caregiving and medical resources (Tahami Monfared et al., 2024). Numerous studies indicate that population aging increases the burden for dementia (GBD 2019 Collaborators, 2021; Li et al., 2022), and the strongest risk factor of dementia is advanced age (Jia et al., 2020).

ADOD is a multifaceted and complex disorder, influenced by a myriad of risk factors spanning physical health, lifestyle choices, medical history, socioeconomic status, psychosocial factors, and environmental conditions. This process can last for several decades (Galvin and Sadowsky, 2012). Given the lack of effective measures to block or even reverse the course of the disease, interventions and prevention targeting risk factors are of great significance in reducing the burden of ADOD (Jack et al., 2018; Yang et al., 2023).

At present, the COVID-19 pandemic has shifted global health priorities to controlling the transmission of SARS-CoV-2 and responding to the added demands on health services (GBD 2021 Causes of Death Collaborators, 2024; GBD 2021 Diseases and Injuries Collaborators, 2024). Over the past three decades, numerous studies have highlighted the substantial global disease burden associated with the ADOD. However, the impact of the COVID-19 pandemic on this burden remains unknown.

To address this gap, we conducted an updated assessment of trends in ADOD, examining prevalence, incidence, mortality, and disability-adjusted life years (DALYs) from 1990 to 2021 at the global, regional, and national levels, with a particular focus on the COVID-19 pandemic. The findings of this study will provide critical theoretical support for policies and interventions aimed at reducing the disease burden, and contribute to the development of more public health strategies for ADOD.

## Materials and methods

### Data source

The data used in this study were sourced from the GBD 2021, a comprehensive dataset that includes information on incidence, prevalence, and mortality across age and sex for over 371 diseases and injuries in 204 countries and territories. The sociodemographic index (SDI), a composite measure ranging from 0 to 1, is calculated based on factors such as educational attainment, economic conditions, and the total fertility rate of a country or region. The 204 countries and territories were classified into five SDI categories: High SDI (>0.81), High-middle SDI (0.70–0.81), Middle SDI (0.61–0.70), Low-middle SDI (0.46–0.61), and Low SDI (< 0.46) (Yang et al., 2024). Differences by sex and age groups were also examined. To eliminate age-related confounding factors, age-standardized rates (ASRs) were applied in the analysis for consistency. Temporal trends of various rates were assessed using the estimated annual percentage change (EAPC) model: ln (val) = b × year + a, where “val” represents the age-standardized rate value, b is the year coefficient, a is the intercept, and year denotes the calendar year. An EAPC > 0 with a 95% UI > 0 indicates an increasing trend in ASR, whereas EAPC < 0 with a 95% UI < 0 signifies a decreasing trend (Boyle et al., 2019). If neither condition was met, the ASRs were considered to be stable over time. Population data were obtained from the United Nations World Population Prospects 2022 Revision,^1^ which provides global population projections from 1950 to 2100, categorized by sex and age. This study utilized publicly available data, so ethical approval was not required.

### Statistical analysis

This study focused on patients with ADOD aged ≥ 40 years and older, as there were no cases of ADOD in individuals aged <40 years. We reported the total numbers along with their corresponding 95% uncertainty intervals (UI), examining trends in the prevalence, incidence, mortality, and DALYs associated with ADOD, along with their age-standardized rates (ASRs) and respective 95% UIs. These trends were analyzed by sex, age, year, SDI subregions, GBD regions, and 204 countries and territories. In the calculation of 95% UIs, descending trends were indicated if the upper limit was less than 0, and ascending trends were shown if the lower limit was greater than 0. All statistical analyses were performed using R software (Version 4.3.1, MathSoft, Cambridge, MA, United States), with a *P*-value of < 0.05 considered statistically significant.

## Results

### Globally

In 2021, 56.9 (95% UI: 49.4, 65.0) million individuals lived with ADOD globally, with an age-standardized rate of 694 (95% UI: 602.9, 794.1) per 100,000 and an increase of 3.2% (95% UI: 1.7, 4.2) since 1990.

The number of new cases of ADOD was reached 9.8 (95% UI: 8.6, 11.2) million in 2021, and the ASIR was 119.8 (95% UI: 105, 135.9) per 100,000, exhibited an increase of 2.4% (95% UI: 1, 3.3) from 1990 (Table 1). Over the study period, the ASIR showed a slight downward trend (EAPC, -0.02, 95% UI: -0.04, 0).

**TABLE 1:** Disease burden of AD stratified by global, regional and national level from 1990 to 2021.

	Prevalence			Incidence			Death			DALYs		
Location	Number (*10^∧^5)	ASRs(per 100,000)	Percentage change in ASRs from 1990 to 2021	Number (*10^∧^3)	ASRs(per 100,000)	Percentage change in ASRs from 1990 to 2021	Number (*10^∧^3)	ASRs(per 100,000)	Percentage change in ASRs from 1990 to 2021	Number (*10^∧^3)	ASRs(per 100,000)	Percentage change in ASRs from 1990 to 2021
Global	568.6 (493.8,649.8)	694 (602.9,794.1)	3.2 (1.7,4.2)	9837.1 (8620.5,11163.7)	119.8 (105,135.9)	2.4 (1,3.3)	1952.7 (513,4984.7)	25.2 (6.7,64.2)	0.5 (-3.8, 7)	36332.7 (17237.6, 76873.3)	451 (212.7, 950.2)	1.2 (-2.8, 5.1)
**GBD regions**												
High-income Asia Pacific	41.1 (35.5, 46.9)	684.8 (597, 780.1)	4.1 (1.9, 6.1)	701.8 (614.6, 802.7)	118.6 (103.4, 135)	1.4 (−0.4, 3.3)	200.2 (57.9, 463.2)	26.6 (7.5, 63)	−4.3 (−11.5, 4)	3028.7 (1439.6, 6015.5)	461.3 (222.4, 928.3)	−1.9 (−8.9, 4)
High-income North America	55.1 (48, 63)	775.1 (673.7, 885.8)	−5 (−6.8, −3.7)	928.3 (812.6, 1051.3)	131.4 (114.7, 149)	−5.9 (−7.5, −4.6)	216.7 (58.3, 541.3)	28.2 (7.5, 71.3)	−2.6 (−5.2, 0.5)	3664.5 (1728.3, 7650.5)	499.2 (236.4, 1041.6)	−4.4 (−6.2, −2.7)
Western Europe	77.5 (67.1, 88.1)	670.4 (583.8, 762.6)	−3 (−5.9, 0.1)	1352.8 (1182.3, 1543.9)	118.6 (103.4, 134.2)	−3.2 (−6, −0.1)	339.6 (90.9, 836.3)	25.8 (6.8, 64)	−3.7 (−8.2, 1.9)	5385.9 (2539.4, 11019)	443.2 (211.9, 909.9)	−3.7 (−7.3, −0.4)
Australasia	3.6 (3.2, 4.1)	604.4 (526.8, 679.9)	−14.5 (−19.2, −10.3)	63.3 (55.5, 71.3)	105.4 (92.6, 118.6)	−14.8 (−19.6, −10.2)	15.3 (4, 38.6)	23.2 (6, 58.7)	−5.5 (−10.3, 1.3)	252.2 (117.6, 522.8)	405.1 (190.5, 836.3)	−8.7 (−13, −5.3)
Andean Latin America	2.5 (2.2, 2.8)	444.1 (384.1, 507.7)	−1.5 (−4.6, 1.1)	44.5 (38.6, 50.8)	79.5 (68.9, 91)	−1.3 (−4.4, 1.3)	7.7 (1.9, 20.7)	14.1 (3.5, 37.9)	−3.2 (−16.1, 14.7)	151 (72.7, 318.4)	272 (131.1, 573.6)	−2.3 (−12.1, 8.7)
Tropical Latin America	18.6 (16.2, 21.2)	759.8 (660, 867.5)	0.2 (−2, 2.5)	311.6 (274.8, 352.7)	126.8 (111.8, 144.4)	−1.8 (−3.7, 0.3)	65.2 (17.1, 164.4)	27.3 (7.1, 68.9)	−2.8 (−6.9, 2)	1226.2 (576.5, 2613.8)	503.2 (235.7, 1070.8)	−1.4 (−4.8, 1.6)
Central Latin America	14 (12.2, 15.9)	596.5 (519, 680.6)	−4.7 (−6.2, −3.3)	248.7 (218.3, 282.1)	106.1 (92.5, 121)	−4.6 (−5.9, −3.4)	38.7 (9.6, 102.5)	16.8 (4.2, 44.6)	−1.8 (−9.3, 7.9)	783.9 (384.3, 1643.5)	335.6 (163.8, 703.5)	−2.2 (−7.5, 3.4)
Southern Latin America	5.5 (4.7, 6.2)	595.3 (514.5, 676.4)	−4.4 (−7.1, −1.8)	98.3 (85, 112.6)	107.1 (92.9, 122.4)	−4.2 (−6.6, −1.7)	18.8 (4.8, 49)	20.1 (5.1, 52.3)	−2.6 (−6.9, 3.1)	340.2 (162.5, 709.4)	368.2 (176.2, 766.7)	−2.9 (−5.8, 0.3)
Caribbean	3 (2.6, 3.4)	550.2 (476.9, 624.8)	−1.3 (−3.8, 1.5)	52.4 (46, 59.4)	95.6 (83.5, 108.6)	−2.1 (−4.3, 0.2)	9.1 (2.2, 24.6)	15.8 (3.9, 43.2)	−3.3 (−11.5, 6.7)	174.2 (85.2, 368.5)	313.6 (153.6, 661.2)	−2.5 (−8.2, 2.9)
Central Europe	15.4 (13.3, 17.7)	641.2 (554.1, 732.3)	−2.4 (−3.6, −1.2)	269.7 (233.1, 309.8)	112.4 (98, 128.5)	−2.3 (−3.4, −1.1)	49.9 (12.5, 133.2)	20.5 (5.2, 54.3)	−1.4 (−5.6, 4.1)	933.2 (446, 1970.6)	386 (184.2, 812.2)	−1.5 (−4.6, 1.9)
Eastern Europe	23.8 (20.6, 27.3)	658.7 (571.3, 752.7)	−1.6 (−2.8, −0.2)	416.1 (361.6, 476.9)	115.7 (100.9, 131.9)	−1.4 (−2.6, −0.1)	75.3 (18.3, 209.1)	21 (5.1, 57.8)	−2.4 (−8.2, 5.8)	1438.7 (673.9, 3104.9)	396.7 (186.4, 853.8)	−1.7 (−6.1, 3.3)
Central Asia	4 (3.5, 4.5)	626.8 (541.9, 713.5)	−1.8 (−3.4, −0.3)	69.8 (61.2, 79.1)	109.8 (95.9, 125.3)	−1.7 (−3.3, −0.3)	11 (2.7, 30.4)	20.1 (5, 55.7)	−2.9 (−10, 4.1)	232.3 (112.6, 498.3)	379.2 (181.2, 819.1)	−2.4 (−7.5, 2.6)
North Africa and Middle East	26.8 (23.2, 30.4)	772.7 (671.2, 877.6)	−4.9 (−6.2, −3.7)	468 (411.5, 531)	132.2 (115.8, 150.4)	−4.3 (−5.5, −3.2)	73.8 (18.1, 190.5)	25.6 (6.3, 66.8)	−8.6 (−14.1, −2.4)	1566.1 (753.7, 3314.9)	476.3 (225.6, 1004.2)	−7.7 (−11.7, −3.4)
South Asia	51.5 (44.4, 58.9)	437.1 (377, 501)	−2.1 (−3.3, −1)	924.8 (800, 1056.3)	79 (68.3, 90.5)	−1.9 (−3.1, −1.1)	165.4 (39.4, 454.1)	17.2 (4.1, 47.3)	21.9 (10.1, 39.2)	3450.1 (1531.6, 7711.4)	308.3 (135.5, 685)	13.3 (4.5, 23.2)
Southeast Asia	33.9 (29.2, 38.6)	644.4 (560.6, 737.7)	−4.5 (−5.8, −3.4)	578.2 (505.9, 658.5)	110.1 (96.1, 125.7)	−4.2 (−5.3, −3)	98.4 (24.2, 259.3)	22.6 (5.6, 59.2)	9.6 (−1.4, 26)	2086.2 (973.5, 4362.2)	418.6 (193.3, 883.7)	4.6 (−3, 14.1)
East Asia	174.1 (148.5, 201.4)	887.9 (759.9, 1027.5)	27.3 (23.8, 30.4)	2988.7 (2569.2, 3434.4)	149.6 (129.6, 171.1)	24.4 (20.7, 27.4)	507.7 (129.2, 1368.5)	30.4 (7.8, 81.3)	−1.5 (−15.1, 18.2)	10359.1 (5080.4, 22833.7)	555.1 (267.6, 1222.9)	5.3 (−8.6, 21.5)
Oceania	0.3 (0.3, 0.3)	644.8 (554.3, 737.4)	−4.7 (−6.8, −2.7)	5.4 (4.7, 6.2)	112 (97, 128.8)	−4.4 (−6.4, −2.4)	0.7 (0.2, 2)	21.1 (5.2, 59.9)	−8.1 (−19.3, 5.2)	17.6 (8.4, 38.1)	397.8 (184.8, 863.9)	−7 (−15.4, 2.4)
Western Sub-Saharan Africa	5.7 (4.9, 6.5)	406 (352.9, 462.4)	−7.1 (−8, −6.3)	102 (88.9, 115.1)	73.2 (63.4, 83.5)	−6.7 (−7.6, −6.1)	20 (4.7, 56.3)	19.3 (4.7, 53.6)	6.9 (−4.7, 21.7)	407.6 (181.2, 942.5)	320.7 (138.3, 745.1)	2.9 (−6.1, 13)
Eastern Sub-Saharan Africa	6.9 (6, 7.8)	588.7 (513.9, 667.2)	−4.8 (−6.1, −3.6)	121.2 (106.4, 137.3)	102.4 (89.7, 116.1)	−4.4 (−5.5, −3.4)	23.3 (5.6, 64.4)	27.2 (6.7, 73.3)	12.5 (2.2, 27.7)	488.1 (218.5, 1080.9)	460.7 (200.7, 1028.3)	7 (−0.9, 16.9)
Central Sub-Saharan Africa	2.5 (2.2, 2.9)	750.5 (654.7, 848.1)	−0.3 (−3.1, 2.3)	44.1 (38.6, 49.6)	126.1 (111.3, 143.2)	−0.6 (−3, 1.8)	8.1 (1.9, 22.2)	34.9 (8.4, 93.2)	14.8 (−4, 38.4)	178.4 (78.7, 408.1)	591.4 (255.7, 1360.3)	10.5 (−3.4, 28.2)
Southern Sub-Saharan Africa	2.7 (2.3, 3.1)	606.7 (524.7, 692.1)	−5.2 (−6.7, −3.9)	47.4 (41.3, 54)	107.1 (93.2, 122.2)	−5 (−6.3, −4)	7.9 (1.9, 21.8)	22.7 (5.4, 62.8)	5.1 (−3.4, 16.8)	168.4 (77.8, 376.4)	409 (187.4, 904.6)	1.7 (−4.7, 10.2)
**Sociodemographic index**												
Low SDI	18.5 (16, 21)	514.4 (447.2, 584.2)	−4.7 (−5.7, −3.7)	328.7 (287.1, 373)	90.9 (79, 103.1)	−4.4 (−5.3, −3.5)	58.6 (13.9, 162.6)	22.1 (5.3, 61.3)	12.8 (3.9, 26.3)	1260.6 (565, 2856.2)	383 (167.7, 863.7)	6.9 (−0.2, 14.8)
Low-middle SDI	60.1 (52.1, 68.4)	524.5 (455.8, 596.8)	−3.3 (−4.2, −2.6)	1063.3 (929.6, 1207.8)	92.6 (80.8, 105.7)	−3.1 (−3.9, −2.4)	189.6 (45.9, 510.2)	20 (4.9, 54.1)	12.9 (5.2, 23.7)	3925.3 (1774.1, 8561.5)	360.4 (164.1, 783.9)	7.6 (1.4, 14.6)
Middle SDI	168 (144.9, 193.1)	723.4 (623.3, 830.9)	11 (9, 12.6)	2902.1 (2542.3, 3314.8)	123.8 (108.2, 141.3)	9.3 (7.1, 10.9)	493.4 (123.1, 1282.1)	24.6 (6.3, 64.5)	3.7 (−4.6, 15.7)	10141.2 (4901.2, 21854.8)	455.3 (216, 982.8)	4.8 (−2.3, 12.9)
High-middle SDI	149.3 (128.6, 171.6)	766.2 (659.8, 879.6)	11.8 (10.2, 13.3)	2582.3 (2252, 2941.8)	132.4 (115.4, 150.8)	11.8 (10.3, 13.1)	490.1 (128.6, 1265.1)	26.4 (7, 68.3)	2 (−4.9, 11.2)	9243.8 (4399.6, 19638.4)	481.7 (228.8, 1024)	4.5 (−2.2, 11.6)
High SDI	172.2 (150.3, 195.5)	709.5 (619.9, 807.4)	−2 (−3.7, −0.4)	2952.1 (2586.7, 3344.5)	122.6 (107.5, 138.4)	−3.6 (−5.3, −2.1)	719.3 (196.7, 1762.4)	26.2 (7.1, 64.8)	−3.2 (−7.6, 2.1)	11732 (5566.8, 24033.2)	460.8 (220.4, 948.4)	−3 (−6.3, −0.4)

In terms of mortality, ADOD accounted for 1.9 (95% UI: 0.5, 4.9) million deaths in 2021, with an age-standardized rate of 25.2 (95% UI: 6.7, 64.2) per 100,000, and an increase of 0.5% (95% UI: -3.8, 7) since 1990. The EAPC in ASDR was 0.02 (95% UI: 0, 0.03), suggesting a gradual upward trend in mortality.

In 2021, the age standardized rate, measured with disability-adjusted life years (DALYs), reached 451 (95% UI: 212.7, 950.2) per 100,000, accompanied by an EAPC of -0.02 (95% UI: -0.03 to -0.01), indicating a downward tendency in disease burden (Tables 1, 2).

**TABLE 2:** EAPC in the ASR of prevalence, incidence, death and DALYs from 1990 to 2021.

	EAPC			
Location	ASR of prevalence	ASR of incidence	ASR of death	ASR of DALYs
Global	0 (−0.02, 0.03)	−0.02 (−0.04, 0)	0.02 (0, 0.03)	−0.02 (−0.03, −0.01)
**21GBD regions**				
High−income Asia Pacific	0.28 (0.23, 0.33)	0.19 (0.14, 0.24)	−0.12 (−0.14, −0.1)	0.01 (−0.01, 0.04)
High-income North America	−0.21 (−0.23, −0.19)	−0.22 (−0.24, −0.21)	−0.06 (−0.08, −0.04)	−0.18 (−0.19, −0.16)
Western Europe	−0.11 (−0.14, −0.08)	−0.14 (−0.18, −0.11)	−0.05 (−0.07, −0.04)	−0.12 (−0.13, −0.11)
Australasia	−0.54 (−0.58, −0.51)	−0.56 (−0.6, −0.52)	−0.13 (−0.15, −0.11)	−0.3 (−0.32, −0.29)
Andean Latin America	−0.06 (−0.07, −0.05)	−0.06 (−0.07, −0.05)	−0.07 (−0.09, −0.05)	−0.11 (−0.12, −0.09)
Tropical Latin America	−0.05 (−0.09, −0.01)	−0.11 (−0.15, −0.08)	−0.04 (−0.06, −0.03)	−0.05 (−0.07, −0.04)
Central Latin America	−0.11 (−0.12, −0.1)	−0.11 (−0.12, −0.09)	−0.05 (−0.06, −0.04)	−0.07 (−0.07, −0.06)
Southern Latin America	−0.15 (−0.16, −0.14)	−0.14 (−0.15, −0.13)	−0.04 (−0.05, −0.02)	−0.08 (−0.1, −0.07)
Caribbean	−0.15 (−0.18, −0.13)	−0.16 (−0.19, −0.14)	−0.09 (−0.1, −0.07)	−0.12 (−0.14, −0.11)
Central Europe	−0.09 (−0.09, −0.08)	−0.08 (−0.09, −0.07)	−0.02 (−0.03, −0.01)	−0.05 (−0.06, −0.04)
Eastern Europe	−0.08 (−0.12, −0.04)	−0.08 (−0.11, −0.04)	−0.04 (−0.06, −0.03)	−0.07 (−0.09, −0.05)
Central Asia	−0.06 (−0.07, −0.04)	−0.06 (−0.07, −0.04)	−0.08 (−0.09, −0.07)	−0.1 (−0.11, −0.09)
North Africa and Middle East	−0.17 (−0.17, −0.16)	−0.14 (−0.15, −0.13)	−0.21 (−0.26, −0.17)	−0.27 (−0.3, −0.25)
South Asia	−0.18 (−0.22, −0.14)	−0.18 (−0.22, −0.14)	0.68 (0.65, 0.71)	0.4 (0.37, 0.43)
Southeast Asia	−0.14 (−0.16, −0.12)	−0.14 (−0.15, −0.12)	0.43 (0.37, 0.49)	0.12 (0.08, 0.15)
East Asia	0.43 (0.35, 0.51)	0.4 (0.33, 0.47)	−0.14 (−0.17, −0.11)	−0.02 (−0.06, 0.02)
Oceania	−0.2 (−0.22, −0.18)	−0.18 (−0.2, −0.16)	−0.21 (−0.25, −0.17)	−0.28 (−0.3, −0.26)
Western Sub-Saharan Africa	−0.24 (−0.26, −0.22)	−0.23 (−0.24, −0.21)	0.27 (0.24, 0.29)	0.14 (0.1, 0.18)
Eastern Sub-Saharan Africa	−0.14 (−0.15, −0.14)	−0.13 (−0.14, −0.12)	0.38 (0.37, 0.39)	0.24 (0.23, 0.25)
Central Sub-Saharan Africa	0 (−0.02, 0.02)	−0.01 (−0.04, 0.01)	0.45 (0.41, 0.49)	0.36 (0.33, 0.4)
Southern Sub-Saharan Africa	−0.14 (−0.15, −0.13)	−0.14 (−0.15, −0.12)	0.3 (0.24, 0.37)	0.05 (0, 0.1)
**Sociodemographic index**				
Low SDI	−0.19 (−0.21, −0.18)	−0.19 (−0.2, −0.17)	0.32 (0.26, 0.38)	0.24 (0.18, 0.29)
Low-middle SDI	−0.17 (−0.19, −0.15)	−0.17 (−0.18, −0.15)	0.43 (0.42, 0.44)	0.23 (0.22, 0.24)
Middle SDI	0.12 (0.08, 0.17)	0.09 (0.05, 0.14)	0.08 (0.06, 0.1)	0.03 (0, 0.05)
High-middle SDI	0.21 (0.17, 0.25)	0.22 (0.18, 0.26)	0.07 (0.05, 0.09)	0.07 (0.04, 0.09)
High SDI	−0.05 (−0.06, −0.04)	−0.11 (−0.12, −0.1)	−0.08 (−0.09, −0.06)	−0.11 (−0.12, −0.1)

### Regionally

Our findings indicate that East Asia regions bear the heaviest burden of ADOD. In detail, the ASPR were highest in East Asia, reaching 887.9 (95% UI: 759.9, 1027.5) per 100,000 population. This figure was 2.19 times that of Western Sub-Saharan Africa, which exhibits the lowest prevalence rate (406 per 100,000; 95% UI: 352.9, 462.4). Over the same period from 1990 to 2021, the ASPR of ADOD in East Asia increased by 27.3% (95% UI: 23.8, 30.4), accompanied by an EAPC of (95% UI: 0.35, 0.51), representing the highest increase all over the world and greatly exceeding the global average increase (Table 2). And East Asia recorded the highest ASIR, 149.6 (95% UI: 129.6, 171.1) per 100,000 people, which was 2.04 times higher than that of Western Sub-Saharan Africa. The latter had the lowest ASIR, at 73.2 cases per 100,000 (95% UI: 63.4, 83.5) (Table 1). Meanwhile the ASIR of ADOD in East Asia increased by 24.4% (95% UI: 20.7, 27.4), and the EAPC were 0.4 (95% UI: 0.33, 0.47), representing the highest increase all over the world and far surpassing the global average increase (Table 2).

Our analysis underscores the urgent need for focused attention on the burden of ADOD in Central Sub-Saharan Africa. This region has the highest ASDR globally, at 22.7 (95% UI: 5.4, 62.8) deaths per 100,000 population, positioning it at the forefront of the global ADOD mortality landscape. Meanwhile, the ASR of death in South Asia increased by 21.9% (95% UI: 10.1, 39.2) and the EAPC were 0.68 (95% UI: 0.65, 0.71). The ASR of DALYs at Central Sub-Saharan Africa was 591.4 (95% UI: 255.7, 1360.3) per 100,000 people, ranking it the highest globally.

**FIGURE 1:**
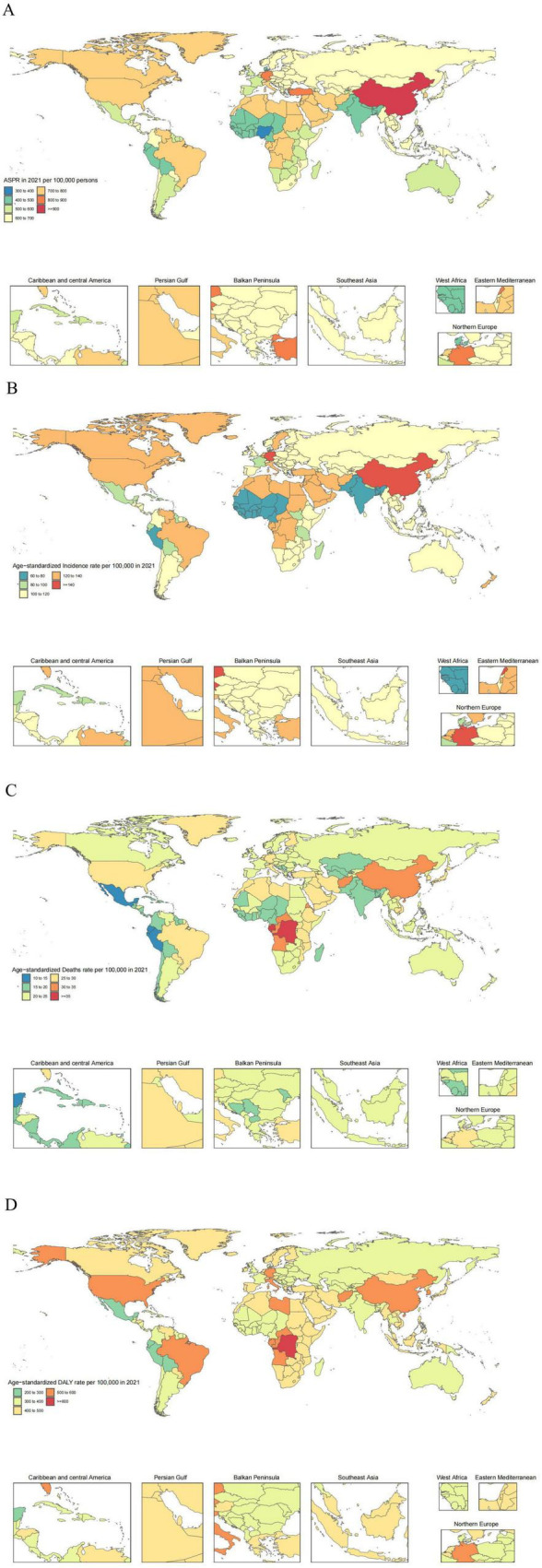
The global disease burden of ADOD for both sexes in 204 countries and territories. **(A)** Prevalence rate. **(B)** Incidence rate. **(C)** Death rate. **(D)** DALYs rate.

Consistent with the regional analysis, we finding that China bear the highest burden of ADOD among the 204 countries and territories, with the highest ASR of prevalence and incidence, 900.8 (95% UI: 770.9, 1043.2) and 151.5 (95% UI: 131.2, 173.3) per 100,000 people, respectively. The same as above, the most significant increases in the ASPR of ADOD were observed in China (EAPC 0.44, 95% UI: 0.36, 0.52) and Taiwan (Province of China) (EAPC 0.46, 95% UI: 0.35, 0.57). Meanwhile, the most obvious growth in the ASIR of ADOD were observed in China (EAPC 0.41, 95% UI: 0.34, 0.49) and Taiwan (Province of China) (EAPC 0.38, 95% UI: 0.29, 0.47) (Figure 1).

**FIGURE 2:**
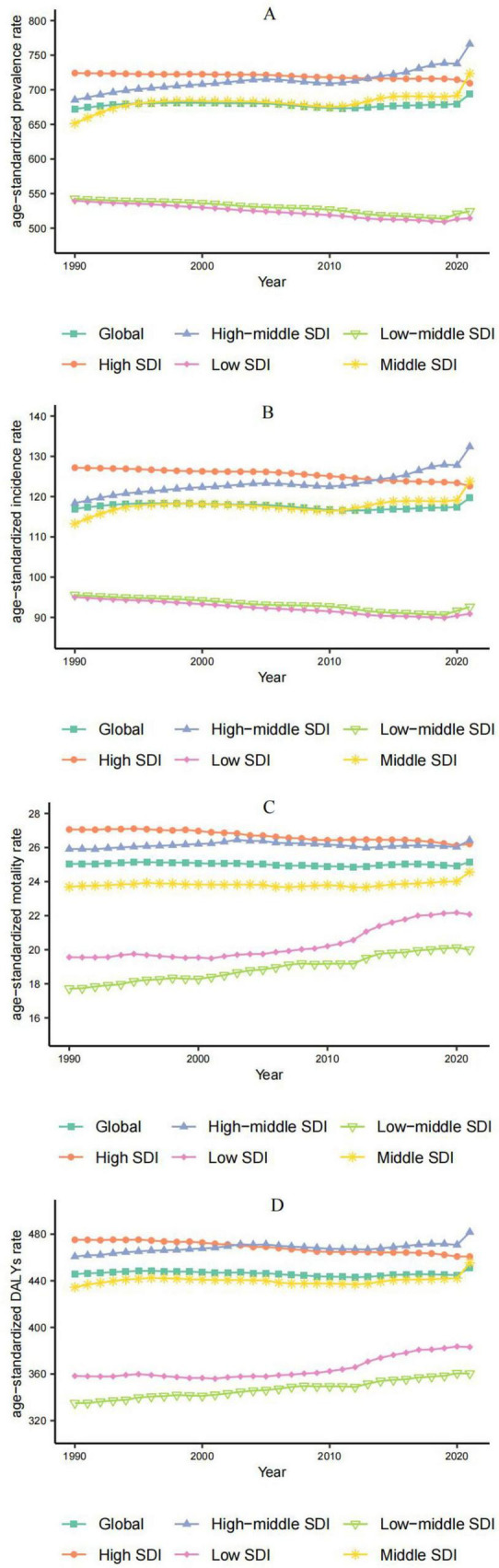
Correlations between the ASR of ADOD and SDI regions in 2021; associations were calculated with Pearson correlation analysis. **(A)** ASR of prevalence. **(B)** ASR of incidence. **(C)** ASR of deaths. **(D)** ASR of DALYs.

When considering the sociodemographic index (SDI), the global burden of ADOD exhibits distinct regional disparities. The ASR of prevalence, incidence, death, and DALYs in ADOD all showed a positive correlation with SDI in the 21 GBD regions in 2021, with ρ values of 0.34, 0.38, 0.16, and 0.17, respectively (Figure 2). High-middle SDI regions experiencing the highest ASPR at 766.2 per 100,000 (95% UI: 659.8, 879.6). Meanwhile, the highest percentage change in ASPR from 1990 to 2021 was occurred in High-middle SDI regions, with the maximum EAPC of 0.21 (95% UI: 0.17, 0.25). Notably, the highest percentage change in ASDR from 1990 to 2021 was occurred in Low-middle and Low SDI regions, with the highest percentage change from 1990 to 2021 being 12.9 (95% UI: 5.2, 23.7) and 12.8 (95% UI: 3.9, 26.3), and the maximum EAPC of 0.43 (95% UI: 0.42, 0.44) and 0.32 (95% UI: 0.26, 0.38), respectively.

However, High SDI regions were the sole areas to demonstrate a downward trend in the ASR of deaths and DALYs from 1990 to 2021, with the EAPC of -0.08 (95% UI: -0.09, -0.06) at ASDR, and -0.11 (95% UI: -0.12, -0.1) at ASR of DALYs, respectively (Tables 1, 2).

### Age and sex

**FIGURE 3:**
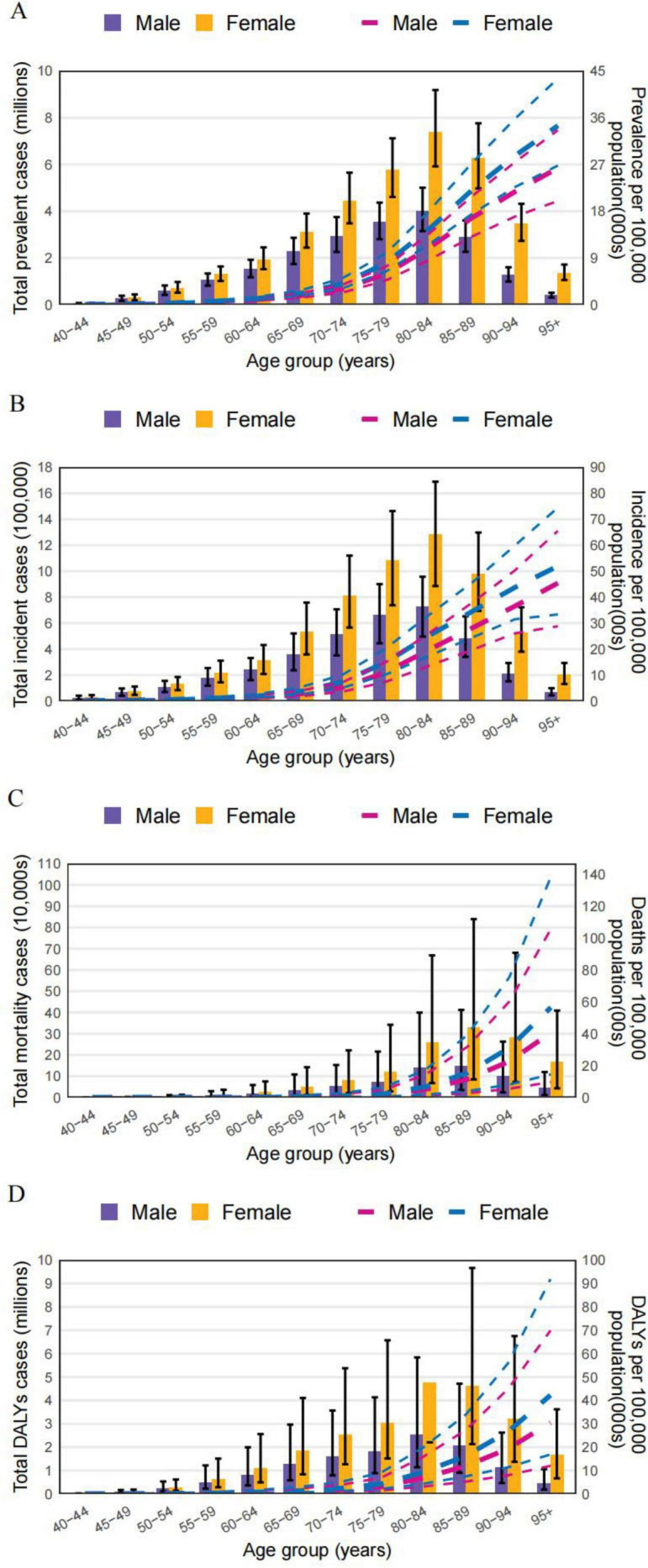
The numbers and age-standardized rate of ADOD among different age groups and sex in 2021. **(A)** Prevalence. **(B)** Incidence. **(C)** Deaths. **(D)** DALYs.

Our findings indicate that the 80–84 age group bears the largest burden of ADOD globally. In detail, the total number of prevalence, incidence and DALYs were most elevated in the 80–84 age group, reaching 11.4 million (95% UI: 9.1, 14.2), 2.0 million (95% UI: 1.4, 2.6), and 7.3 million (95% UI: 3.4, 16.0), respectively. It then decreased with the increasing age. The total number of death was highest in the 85–89 age groups, reaching 0.4 million (95% UI:0.1, 1.1). Meanwhile, the ASR of all measures were started to increase in the 40–44 age group and peaked in the oldest age group (≥95 years) in 2021. Above all, the total cases and the ASR of all measures in women were higher than those in men across all age groups (Figure 3).

**FIGURE 4:**
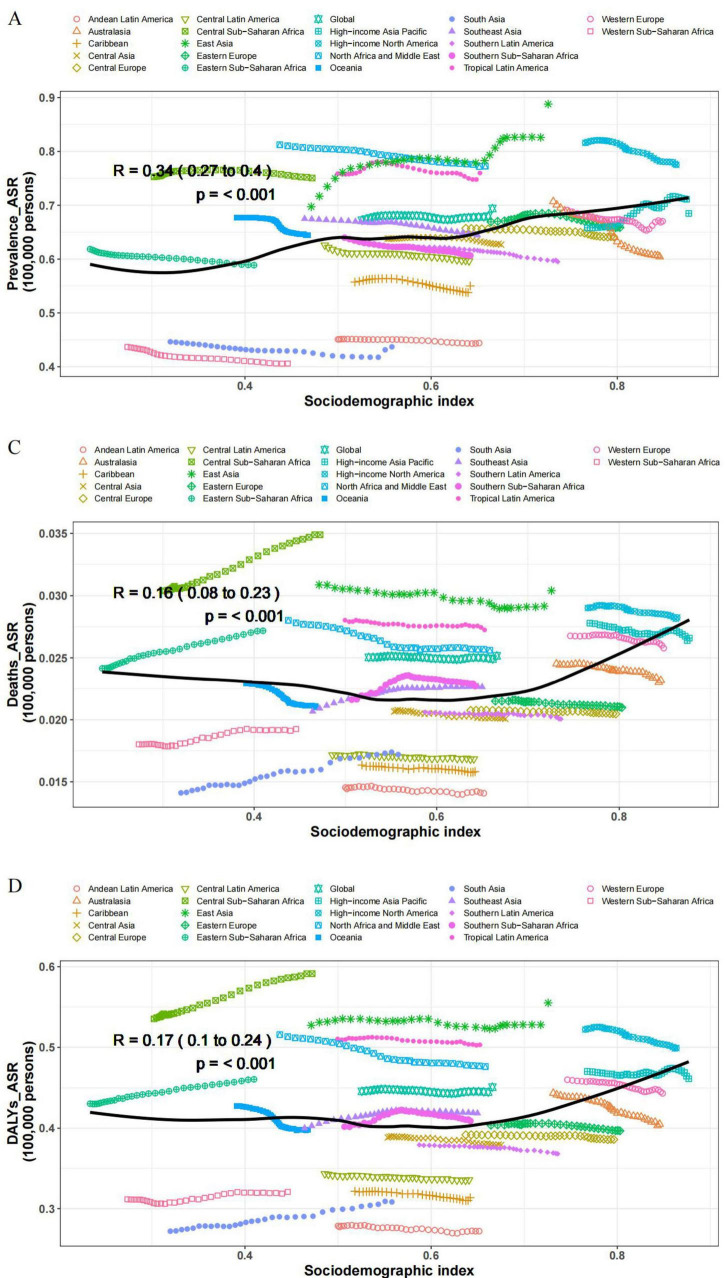
Temporal trend of age-standardized burdens of ADOD from 1990 to 2021. **(A)** ASR of prevalence. **(B)** ASR of incidence. **(C)** ASR of deaths. **(D)** ASR of DALYs.

Over the past three decades, the ASPR of ADOD worldwide has tended to be stable. However, the ASPR of ADOD showed a significant upward trend from 2019 to 2021, which may hace been caused by the global COVID-19 pandemic. Specifically, in different SDI regions, the ASPR of ADOD in the High SDI regions still showed a downward trend from 2019 to 2021, indicating that its medical system was relatively less affected by the impact of the COVID-19 epidemic (Figure 4).

## Discussion

Our current analysis of the global burden of ADOD from 1990 to 2021 highlights the intricate variations and trends across different regions and populations. Worldwide, the incidence, morbidity, mortality, and DALYs associated with ADOD showed substantial increases druing the study period. The slight rise in the age-standardized rate suggests that aging populations play a crucial role in the growing burden of ADOD, which must be addressed when considering strategies to reduce its impact. With the increase in the aging population worldwide, driven by improvements in socioeconomic status, greater access to healthcare, and advances in technology, this aging trend is predicted to continue (Boyle et al., 2018; Cheng and Hu, 2020; Power et al., 2019). The increasing prevalence of age-related diseases, such as cerebrovascular diseases, diabetes, hypertension, obesity, and dyslipidemia, increases the risk of developing ADOD (Sharma et al., 2020; Silva et al., 2019).

Disparities across the SDI regions reveal the dual impact of socioeconomic development on the ADOD burden. High SDI regions exhibited the highest age-standardized rates for prevalence, incidence, mortality, and DALYs. This elevated burden can be attributed to a higher proportion of older adults, more comprehensive case ascertainment, and wider access to clinical diagnoses (Langa, 2015). However, a critical and seemingly paradoxical finding is the observed deceleration in the rate of increase, as indicated by a declining EAPC, in High SDI regions. This trend likely reflects the benefits of superior healthcare quality, effective management of vascular and metabolic risk factors, and a robust social care infrastructure, which may be mitigating the growth rate of the burden despite an aging populace. In contrast, the lower absolute burden estimates in low-SDI regions may partially reflect under-diagnosis and gaps in data availability (Amiri et al., 2025).

Pronounced inter-regional heterogeneity underscores the influence of developmental stage and healthcare resource allocation. East Asia bears the greatest burden of ADOD among all GBD regions, closely linked to its rapid demographic aging, urbanization, and associated lifestyle transitions. The notably lighter burden in the high-income Asia Pacific region highlights the potential for socioeconomic advancement and effective health policies to ameliorate the impact of ADOD (Wang et al., 2023). These nations typically benefit from more mature systems for diagnosis, treatment, and long-term care (Passeri et al., 2022; Zhang et al., 2025). Conversely, the high mortality burden in Central Sub-Saharan Africa starkly exposes profound challenges in healthcare access, fundamental public health infrastructure, and availability of resources for the ADOD prevention and treatment.

Age and sex remain fundamental, non-modifiable determinants of ADOD burden distribution. Our results clearly demonstrate that all age-standardized metrics for ADOD increase markedly with age, peaking in the oldest age groups. This pattern directly confirms that advanced age is the strongest identified risk factor for the disease. The burden of age-related chronic diseases and predisposing genetics will also increase with life expectancy in older adults (He et al., 2025; Rawat et al., 2024). Females consistently carried a higher burden than males across all age groups and geographical settings. This disparity can be explained by a combination of factors, such as reproductive health, hormone levels, genetic predisposition, and mental wellbeing (Meng et al., 2023; Zhu et al., 2021). Women are more prone to structural and functional impairments in the nervous system, which are recognized as risk factors for ADOD. Furthermore, the female brain is naturally more vulnerable to ADOD, partly due to the influence of sex hormones. Additionally, women generally have a longer life expectancy than men, making them a larger segment of the aging population (Bratosiewicz-Wąsik, 2022; Ji et al., 2024). These findings underscore the critical importance of tailoring prevention and intervention strategies for older adults and female populations.

The COVID-19 pandemic, which began in late 2019 and extended through 2021, introduced novel and complex challenges to ADOD management and prevention. Growing evidence suggests that SARS-CoV-2 infection can lead to cognitive impairment and potentially accelerate underlying neuropathological processes (Chen et al., 2020; Kendrick and Isaac, 2021; Sharma, 2021). Beyond direct infection, public health measures such as isolation severely disrupted routine care and social support systems for patients with ADOD, exacerbating psychological distress and behavioral symptoms among both infected and non-infected individuals (Munblit et al., 2022). It has become evident that social isolation and quarantine caused by COVID-19 have had numerous negative effects on patients with ADOD, including increased feelings of loneliness, depression, and anxiety, which, in turn, have resulted in a rapid escalation of behavioral and psychological symptoms (Yu et al., 2020). Furthermore, the restrictions on social interactions during the pandemic have not only posed difficulties for ADOD patients but have also hindered efforts to prevent ADOD (GBD 2021 Nervous System Disorders Collaborators, 2024). Given the chronic nature of ADOD, the long-term consequences of the pandemic on its global burden may take years to fully materialize, suggesting a potential surge in future healthcare demand.

Our study had several limitations that should be considered when interpreting the findings. First, the absence of primary data from some countries, particularly those with low SDI, likely led to an underestimation of the true burden in these regions. Second, the aggregation of all dementia subtypes within the GBD datasets prevents a nuanced analysis of specific etiologies, such as Alzheimer’s disease or vascular dementia, which have distinct risk factors and disease trajectories. Finally, as this analysis includes only the initial 2 years of the COVID-19 pandemic, the long-term impact of the pandemic on cognitive health remains inadequately explored and not yet fully quantified (Wong et al., 2023)Continued surveillance is essential for understanding these evolving dynamics.

## Conclusion

In summary, this study provides insights into the global epidemiological trends of ADOD from 1990 to 2021, including the impact of the COVID-19 pandemic. With an increasing aging population, dementia has become a significant global public health challenge. The distribution of disease burden exhibits distinct variations across regions, sex, and age groups. These insights lead to several targeted recommendations for future research and policy. First, there is an urgent need to develop stratified prevention frameworks that account for regional developmental contexts - enhancing early screening and diagnosis in high-burden areas while prioritizing basic risk factor management in resource-limited settings. Second, our findings underscore the necessity of implementing targeted intervention strategies for women and the elderly population. Finally, establishing a dynamic surveillance network to monitor the long-term neurological impacts of large-scale pandemic events is essential.

## Data Availability

Publicly available datasets were analyzed in this study. This data can be found at: https://vizhub.healthdata.org/gbd-results/.

## Footnotes

1
https://population.un.org/wpp/Download/Standard/Population/
